# Technology-aided assessment of functionally relevant sensorimotor impairments in arm and hand of post-stroke individuals

**DOI:** 10.1186/s12984-020-00748-5

**Published:** 2020-09-25

**Authors:** Christoph M. Kanzler, Anne Schwarz, Jeremia P. O. Held, Andreas R. Luft, Roger Gassert, Olivier Lambercy

**Affiliations:** 1 Rehabilitation Engineering Laboratory, Institute of Robotics and Intelligent Systems, Department of Health Sciences and Technology, ETH Zurich, Zurich, Switzerland; 2 Division of Vascular Neurology and Neurorehabilitation, Department of Neurology, University Hospital Zurich, University of Zurich, Zurich, Switzerland; 3 cereneo, Center for Neurology and Rehabilitation, Zurich, Switzerland; 4 Biomedical Signals and Systems (BSS), University of Twente, Enschede, The Netherlands

**Keywords:** Upper limb assessment, Digital health metrics, Motor control, Neurological disorders

## Abstract

**Background:**

Assessing arm and hand sensorimotor impairments that are functionally relevant is essential to optimize the impact of neurorehabilitation interventions. Technology-aided assessments should provide a sensitive and objective characterization of upper limb impairments, but often provide arm weight support and neglect the importance of the hand, thereby questioning their functional relevance. The Virtual Peg Insertion Test (VPIT) addresses these limitations by quantifying arm and hand movements as well as grip forces during a goal-directed manipulation task requiring active lifting of the upper limb against gravity. The aim of this work was to evaluate the ability of the VPIT metrics to characterize arm and hand sensorimotor impairments that are relevant for performing functional tasks.

**Methods:**

Arm and hand sensorimotor impairments were systematically characterized in 30 chronic stroke patients using conventional clinical scales and the VPIT. For the latter, ten previously established kinematic and kinetic core metrics were extracted. The validity and robustness of these metrics was investigated by analyzing their clinimetric properties (test-retest reliability, measurement error, learning effects, concurrent validity).

**Results:**

Twenty-three of the participants, the ones with mild to moderate sensorimotor impairments and without strong cognitive deficits, were able to successfully complete the VPIT protocol (duration 16.6 min). The VPIT metrics detected impairments in arm and hand in 90.0% of the participants, and were sensitive to increased muscle tone and pathological joint coupling. Most importantly, significant moderate to high correlations between conventional scales of activity limitations and the VPIT metrics were found, thereby indicating their functional relevance when grasping and transporting objects, and when performing dexterous finger manipulations. Lastly, the robustness of three out of the ten VPIT core metrics in post-stroke individuals was confirmed.

**Conclusions:**

This work provides evidence that technology-aided assessments requiring goal-directed manipulations without arm weight support can provide an objective, robust, and clinically feasible way to assess functionally relevant sensorimotor impairments in arm and hand in chronic post-stroke individuals with mild to moderate deficits. This allows for a better identification of impairments with high functional relevance and can contribute to optimizing the functional benefits of neurorehabilitation interventions.

## Introduction

Stroke is a leading cause of acquired adult disability [1]. The incident commonly causes chronic sensorimotor deficits in arm and hand (impairments) [2, 3]. Impairments that are functionally relevant are especially critical for affected individuals, as these impairments reduce the spectrum of activities that an individual can perform (activity limitations) and determine the level of dependence on caregivers. Neurorehabilitation attempts to decrease the level of disability through inter-disciplinary interventions, including physical therapy [4, 5]. Achieving successful rehabilitation, with clear benefits for the independence of individuals typically requires the identification and therapy of functionally relevant impairments [6–8].

Conventional clinical scales are the current standard to evaluate upper limb sensorimotor impairments in research studies and the described impairments mostly show strong links to activity limitations (i.e., functional relevance) [9–13]. However, conventional assessments commonly rely on subjectively rated ordinal scales with ceiling effects that are not sensitive enough to detect fine changes in impairments and even introduce bias when attempting to model sensorimotor recovery [14–16]. Hence, providing a more objective assessment of functionally relevant sensorimotor impairments with sensitive scales should be of primary interest to neurorehabilitation researchers.

Digital health metrics extracted from technology-aided assessments can provide objective and traceable descriptions of upper limb behavior on sensitive, continuous scales without ceiling effects [17–19]. However, the majority of technology-aided assessments focus on characterizing impairments during planar arm movements while providing gravity support [20–23]. This neglects the importance of hand impairments and shadows the effects of certain deficits, such as weakness [19], which are both fundamental when performing daily activities. This questions the functional relevance of these assessments.

More recently, technology-aided approaches started emphasizing the importance of assessing impairments during tasks involving arm movements and hand manipulations without providing arm weight support [24–27]. Such tasks are expected to provide crucial information on fine upper limb impairments in individuals with mild to moderate disability levels and are promising to better identify functionally relevant impairments. However, existing approaches typically rely on time-consuming and complex measurement setups, which reduces their clinical applicability. Further, they mostly focus on kinematic metrics and do not quantify grip force control and its essential role in daily life activities [28, 29]. Also, the clinimetric properties of such digital health metrics are often insufficiently evaluated, thereby challenging their interpretability and acceptability as clinical endpoints [17, 30].

The Virtual Peg Insertion Test (VPIT) addresses many of the limitations of existing technology-aided assessments by recording movement and grip force patterns during a virtual goal-directed manipulation task requiring coordinated arm and hand movements [31, 32]. Previous research indicated the feasibility of the approach in neurologic individuals with mild to moderate sensorimotor impairments [32–35]. In addition, ten digital health metrics capturing sensorimotor impairments have been established for the VPIT and allowed for an accurate discrimination between neurologically intact and affected individuals [32]. However, whether the VPIT metrics provide a multi-dimensional evaluation of impairments in arm and hand that are functionally relevant has not been evaluated yet. Further, the clinimetric properties (test-retest reliability, measurement error, learning effects, concurrent validity) of the VPIT metrics have mainly been evaluated in unaffected subjects, thereby leaving their applicability and robustness in post-stroke individuals unexplored.

The objective of this work was to evaluate the ability of the digital health metrics from the VPIT to characterize arm and hand sensorimotor impairments that are relevant for performing functional tasks, by evaluating their clinimetric properties in 30 chronic post-stroke subjects.

## Methods

### Virtual Peg Insertion Test (VPIT)

**Fig. 1:**
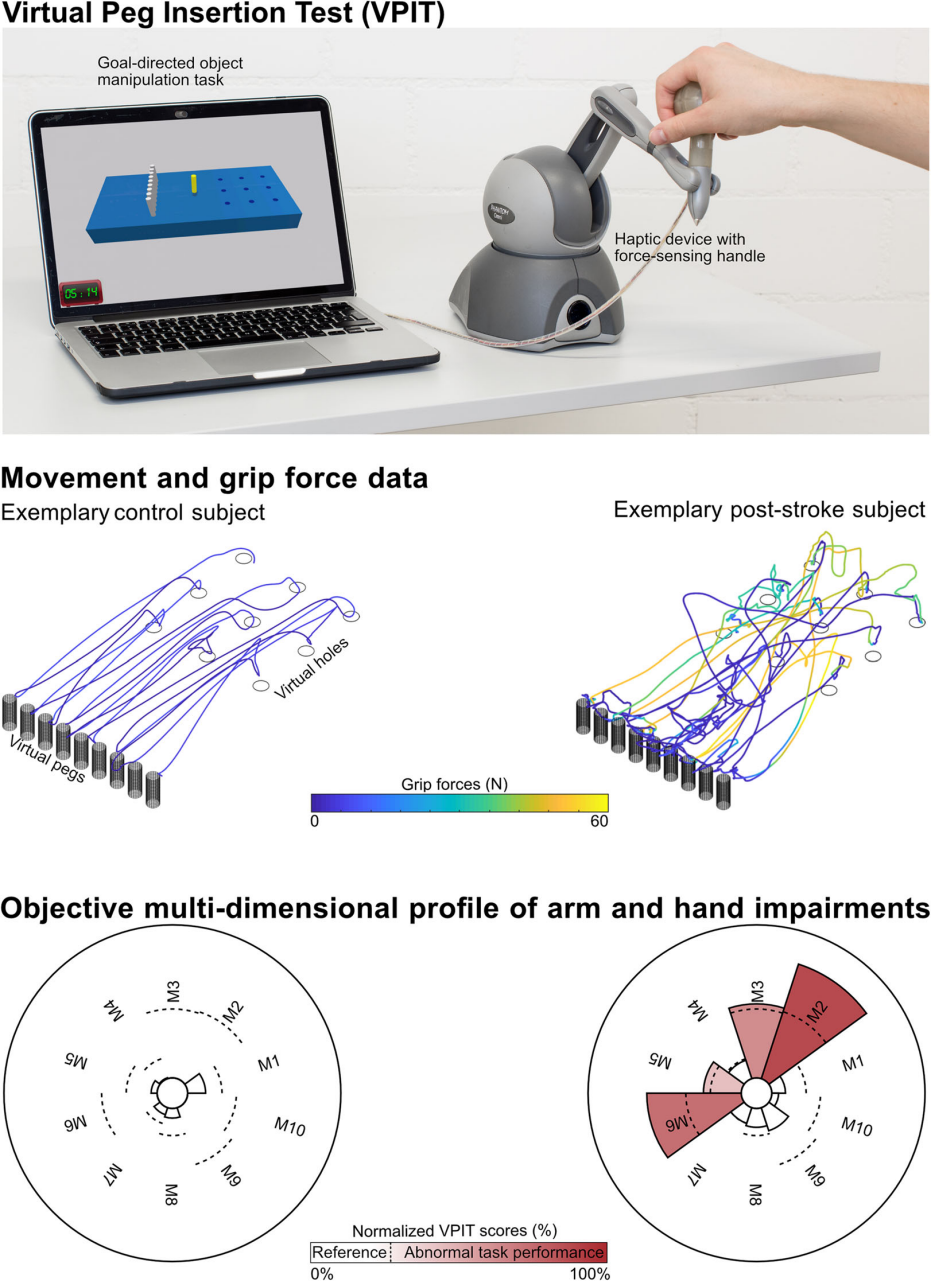
Concept of the Virtual Peg Insertion Test (VPIT). Visualization of hardware setup (top), extracted movement and grip force data (middle) for one exemplary control (age 36 yrs, male) and post-stroke (age 52 yrs, male, FMA-UE 55, ARAT 52) subject, and the processed impairment profiles (bottom) relying on 10 metrics (M1-M10). M1: log jerk transport. M2: log jerk return. M3: SAL return. M4: path length ratio transport. M5: path length ratio return. M6: velocity max return. M7: jerk peg approach. M8: force peaks transport. M9: force rate SAL transport. M10: force rate SAL hole approach. SAL: spectral arc length

The VPIT (Fig. 1, video at https://youtu.be/TyJyd5uVN68) as an upper limb sensorimotor assessment has been described in detail in previous work [31–33]. In short, it consists of a commercial haptic end-effector device (PhantomOmni or Geomagic Touch, 3D Systems, USA), a rapid-prototyped grasping force sensing handle, and a virtual reality environment on a personal computer (total material costs approximately 4000 USD). The virtual reality environment displays a virtual pegboard task that requires the insertion of nine virtual pegs into nine holes. The pegboard has dimensions similar to the Nine Hole Peg Test (26.8 ×12.8 ×6.2 cm) [36]. More specifically, a virtual cursor can be controlled through the coordination of end-effector movements and applied grasping force. To pick up a peg, the cursor first needs to be spatially aligned with the peg. Subsequently, a grasping force of at least 2N has to be maintained to transport the peg towards a hole. The peg can be released in a hole upon a reduction of the grasping force below 2N. Based on this task design, the VPIT engages various aspects of sensorimotor control and assesses goal-directed arm movements, while actively lifting the arm against gravity, in combination with spherical grip force control. Hence, the VPIT should be seen as a hybrid solution, combining elements of the Nine Hole Peg Test (NHPT) and the Box and Block Test (BBT). This is expected to provide a multi-dimensional picture of different sensorimotor impairments in a functional context.

Recently, a core set of 10 kinematic and kinetic VPIT metrics was selected from a set of 77 candidate metrics based on an automated, data-driven metric selection process that optimizes clinically-relevant statistical criteria for longitudinally assessing impairments [32]. These metrics are extracted through an advanced processing and normalization pipeline that is applied to the position and grip force data from the VPIT, sampled at 1 kHz [32]. More specifically, data is low-pass filtered and temporally segmented into the *transport* (gross movement from peg pickup until insertion), *return* (gross movement from peg insertion to next pickup), and *peg approach* (fine movement after return and before transport), *hole approach* (fine movement after transport and before return). Subsequently, metrics were defined for each of these confined phases to quantify different aspects of upper limb sensorimotor impairments in a functional context.

A detailed description of the core set of metrics, their pathophysiological motivation, and mathematical implementation can be found in previous work [32], but is shortly described below for completeness. Smooth movements, represented through a bell-shaped velocity profile, are a hallmark of intact motor control [37]. Movement smoothness was quantified using the normalized logarithmic jerk metric (*log jerk*) calculated during *transport* and *return* as well as the spectral arc length metric of the velocity signal during return (*SPARC return*) [38–40]. Similarly, ballistic movements of unaffected individuals are efficient and tend to follow a trajectory close to the shortest path between start and target. Movement efficiency was characterized using the *path length ratio* (shortest possible distance divided by the actually covered distance) during *transport* and *return* [41]. Movement speed was quantified using the maximum velocity during *return* (*velocity max. return*) and the endpoint-precision of the ballistic movement using the jerk metric calculated during the peg approach (*jerk peg approach*). Further, three metrics describing the smoothness of grip force coordination during different movement phases were defined. This included the number of peaks in the grip force rate (first time-derivative of grip force) during *transport* (*grip force rate num. peaks transport*). Additionally, the SPARC was applied to grip force rate data recorded during *transport* (*grip force rate SPARC transport*) and *hole approach* (*grip force rate SPARC hole approach*). The clinimetric properties of all ten metrics have been positively evaluated in neurologically intact subjects, which indicated that the metrics have high test-retest reliability, low measurement error, and do not show systematic learning effects [32]. In addition, all metrics showed strong discriminative ability between a normative reference population and a group of 89 neurologically affected subjects, thereby demonstrating their ability to capture sensorimotor impairments [32].

For all metrics, mixed effect models were generated to compensate for confounding factors such as age, gender, tested body side, and whether the test was performed with the dominant body side or not [32]. Further, the value of each metric was normalized with respect to the median and variability of a reference population containing 120 unimpaired subjects (age 20-80 years, 60 female) that performed the VPIT [32]. Lastly, each metric was additionally normalized with respect to the neurologically affected subject in the VPIT database that completed the VPIT protocol and showed worst performance in a specific metric. This resulted in metrics being defined on an unbounded scale, theoretically ranging from ]−*∞%*,+*∞%*[, with 0% indicating median task performance of the reference population and 100% worst recorded task performance [32].

### Conventional clinical assessments

A battery of conventional clinical assessments were performed to capture the heterogeneity of sensorimotor impairments and activity limitations, and to compare this clinical picture to the one constructed by the VPIT. Individual assessments can take up to 30 minutes to administer [42], which led to extensive sessions well above 60 min to perform the battery of assessments that is presented in the following.

#### Sensorimotor impairments

Motor impairments in hand and wrist as well as flexor/extensor synergies in shoulder, elbow, wrist, and hand were described using the Fugl-Meyer assessment for the upper extremity (FMA-UE, worst score 0, best score 66) [14].

Cognitive impairments were rated with the Montreal cognitive assessment (MOCA), which consists of simple tasks such as drawing, object naming, memory recall, reading, and mathematical operations (0: worst score, 30: best score) [43].

Resistance against passive movements due to increased muscle tone (referred to as spasticity) in shoulder internal rotators, biceps, triceps, wrist flexors and extensors, as well as finger flexors and extensors were defined with the Modified Ashworth Scale (MAS, worst score at 35, best score at 0) that involves the passive movement of the respective joint [44].

Somatosensory impairments of upper arm, lower arm, hand, and finger was measured based on the Erasmus modified Nottingham sensory assessment (EmNSA, worst score 0, best score 40) that focuses especially on tactile sensation, sharp-blunt discrimination, two-point discrimination, and proprioception [45].

#### Activity limitations

The ability to coordinate precise object manipulations with gross arm movements was evaluated with the Action Research Arm Test (ARAT, worst score 0, best score 57), which requires the transfer of small and large items with multiple handgrip types from the bottom to the top of a shelf [46, 47].

Fine manual dexterity was evaluated with the time to insert nine small physical pegs into nine corresponding holes without requiring active lifting of the arm against gravity, as defined by the NHPT [36, 48].

Lastly, gross manual dexterity was reported through the BBT, which requires the transport of as many blocks as possible within one minute across a physical barrier while actively lifting the arm against gravity [47, 49]. For the BBT and NHPT, the outcome measure was normalized with respect to the publicly available reference data to account for the influence of age, gender, and tested body side, as also implemented for the VPIT. More specifically, this was realized by subtracting, for each subject, the mean value of the matched reference data and dividing by the standard deviation of the matched reference data. Hence, a value of 0 indicates mean reference performance, and increasing values indicate an increasing statistical distance to the mean reference performance level.

### Participants and procedures

Thirty post-stroke subjects were recruited at the University Hospital of Zurich (Zurich, Switzerland) and the cereneo, Center for Neurology and Rehabilitation (Vitznau, Switzerland) as part of an observational study (ClinicalTrials.gov Identifier: NCT03135093) that used the VPIT as a secondary outcome next to a battery of clinical assessments focusing on sensorimotor impairments (FMA-UE, MOCA, MAS, EmNSA). The VPIT protocol consisted of receiving standardized instructions, familiarizing with the task by inserting all nine pegs once (data not analyzed), and subsequently performing five repetitions (i.e., inserting all nine pegs five times). The protocol was performed with the most affected and less affected body side, given that both of them might be affected by sensorimotor impairments [50]. The subjects were enrolled into a second measurement session including a repetition of the VPIT protocol and further clinical assessments focusing on activity limitations (BBT, NHPT, ARAT).

All participants gave written informed consent, and all procedures were approved by the local Ethical Committees (ID 2016-02075 and BASEC:2017-00398). Recruited subjects were at least 18 years old with chronic (i.e., at least 6 months ago) ischemic stroke with at least partial ability to lift the arm against gravity and flex and extend the fingers. Exclusion criteria were other concomitant diseases affecting the upper limb, severe sensory deficits, and severely increased muscle tone that considerably limits range of motion.

Participants started the VPIT assessment with the most affected body side and were instructed to perform the task as fast and as precisely as possible. The seated starting position was approximately 45^∘^ shoulder abduction, 10^∘^ shoulder flexion, and 90^∘^ elbow flexion. Subjects received live feedback about the duration of each VPIT repetition through a timer displayed on the computer screen.

### Data analysis

#### Characterization of upper limb sensorimotor impairments and activity limitations

The presence of upper limb impairments was quantified using the ten VPIT metrics and conventional scales. For the VPIT, previously established cut-offs based on the 95^*th*^-percentile of the normative reference population were used to define individuals with abnormal behavior (binary value) in each metric. This dichotomization was only applied for this specific sub-analysis as it allows the abstraction of metrics to the most clinically relevant information, whereas the continuously defined VPIT metrics were used for all other analyses. For the NHPT and BBT, abnormal behavior was defined if task performance was worse than 1.96 times the standard deviation (corresponding to 95^*th*^-percentile) of the publicly available normative reference population [36, 48]. According to the ARAT, activity limitations were present if the score was below 55, as suggested by Hoonhorst et al. [13]. All other conventional scales indicated the presence of impairments if the full score was not reached.

#### Correlation of upper limb sensorimotor impairments with activity limitations

To analyze how both VPIT metrics and conventional impairment scales relate to conventional assessments of activity limitations, Spearman correlation coefficients (*ρ*) were calculated. For the correlation analysis, only data from the most affected side (*ρ*_*ma*_) and the first testing session was included to avoid the influence of ceiling effects in the conventional scales for the less affected body side and learning effects across sessions, respectively. Bonferroni correction was applied for each tested hypothesis to account for multiple comparisons. The intervals suggested by Hinkle et al. were used for interpreting the correlation coefficients: very high: *ρ*_*ma*_≥0.9; high: 0.7 ≤*ρ*_*ma*_<0.9; moderate: 0.5 ≤*ρ*_*ma*_<0.7; low: 0.3 ≤*ρ*_*ma*_<0.5; very low: *ρ*_*ma*_<0.3 [51].

#### Test-retest reliability, measurement error, learning effects, and concurrent validity of VPIT metrics

The evaluation of the clinimetric properties was guided through a previously defined framework for the selection and validation of digital health metrics [32]. More specifically, the repeatability of the VPIT metrics was quantified by their ability to discriminate different subjects across measurement sessions (test-retest reliability) and the measurement error of the task and assessment platform [32, 52, 53]. The former was defined using the intra-class correlation coefficient (ICC A,k). Metrics with an ICC >0.7 passed the evaluation. The latter was characterized using the smallest real difference (SRD), which defines a range of values in which the assessment cannot distinguish between measurement noise and an actual change in the underlying physiological construct. The SRD was defined as $1.96\cdot \sqrt {2} \cdot \sqrt {1-\text {ICC}}$ [54, 55]. The SRD was further normalized (SRD%) with respect to the range of observed values of a metric to enable a comparison across metrics. A previously established cut-off of SRD ≤30.3% was applied to define metrics that have the highest potential to sensitively measure sensorimotor recovery [32]. As the smallest real difference and the corresponding responsiveness of a metric strongly depends on the intra-subject variability, the standard deviation across all repetitions of the VPIT was visualized. In addition, Bland-Altman plots were constructed to inspect systematic errors across test-retest sessions that depend on the range of each metric [56].

Systematic learning effects within and across testing sessions were identified. This is important to distinguish between task-related motor learning and behavioral recovery when using the VPIT to analyze the effect of interventions. In more details, metrics were visualized for each of the five repetitions at test and retest. Subsequently, the slope (*η*) between test and retest for the median across all five repetitions was estimated and normalized with respect to the range of observed values. Strong learning effects were present if a paired t-test indicated significant differences between test and retest and the slope *η* was below or equal -6.35% [32]. When using the metrics as outcome measures in longitudinal studies, metrics with strong learning effects should be avoided.

Lastly, the correlations between conventional impairment scales and the VPIT metrics were calculated, for the most affected body side (*ρ*_*ma*_), to further advance the pathophysiological interpretation of the digital health metrics.

## Results

Out of the 30 recruited post-stroke subjects, the VPIT protocol on the first testing day was completed by 23 and 27 individuals with the most affected and less affected body side, respectively. The reasons for subjects not completing the protocol were: inability to understand the task (1 subject), severe visual deficits (1 subject), severe sensorimotor impairments (less affected side: 1 subject; most affected side: 5 subjects). The age of the 23 subjects that completed the VPIT protocol with the most affected body side was 59.0 (53.5, 68.5) years (median (25^*th*^-percentile, 75^*th*^-percentile)) with 14 of them being female. FMA-UE scores for the most affected (23 subjects) and less affected sides (27 subjects) were 49 (41, 57) and 65 (63, 66) respectively. ARAT scores for the most affected and less affected sides were 47 (39, 55) and 57 (57, 57), respectively. Detailed subject characteristics can be found in Table SM4.

Twenty-one subjects also participated in the retest protocol, with 18 and 21 successfully completing it with the most affected and less affected side, respectively. The time between test- and retest was 7.9 (5.2, 16.1) days. The time to administer the VPIT protocol (instructions, familiarization, and five repetitions) was 16.7 (12.3, 26.0) min and 10.0 (7.9, 16.0) min for the most affected and less affected side, respectively, during the first testing session.

### Characterization of sensorimotor impairments and activity limitations

The presence of sensorimotor impairments and activity limitations on a population level can be found in Table 1. According to the defined criteria, the percentage of subjects with sensorimotor impairments on the most affected and less affected sides varied between 70.0%-100.0% and 9.1%-50.0%, respectively, depending on the conventional scale. Similarly, the percentage of subjects with activity limitations ranged from 65.0%-90.0% and 4.5%-54.5% for the most affected and less affected side, respectively. Depending on the metric, the VPIT indicated sensorimotor impairments in 10.0%-50.0% and 0.0%-31.8% of all individuals with the most affected and less affected side, respectively. In total, 90% and 50% of all individuals showed impairment in at least one VPIT metric with the most affected and less affected side, respectively.

**Table 1:** Characterization of impairments and activity limitations

	**Percentage of subjects with disability**	
	Most affected side	Less affected side
	*n* = 20	*n* = 22
**Conventional scales: impairments**		
FMA-UE	100.0%	50.0%
MAS	75.0%	9.1%
EmNSA	70.0%	18.2%
**Conventional scales: activity**		
BBT	90.0%	54.5%
ARAT	70.0%	4.5%
NHPT	70.0%	9.1%
**VPIT: impairments in activity context**		
Log jerk transport	45.0%	8.2%
Log jerk return	35.0%	9.1%
SPARC return	30.0%	9.1%
Path length ratio transport	45.0%	4.5%
Path length ratio return	35.0%	13.6%
Velocity max. return	50.0%	31.8%
Jerk peg approach	30.0%	0.0%
Grip force rate num. peaks transport	50.0%	22.7%
Grip force rate SPARC transport	10.0%	9.1%
Grip force rate SPARC hole approach	45.0%	4.5%

MAS: Modified Ashworth Scale; NHPT: Nine Hole Peg Test; EmNSA: Erasmus modifications to the Nottingham Sensory Assessment; BBT: Box and Block Test; ARAT: Action Research Arm Test; FMA-UE: Fugl-Meyer Assessment Upper Extremity.

Conventional assessments and the VPIT were used to define the presence of sensorimotor impairments and activity limitations. For the VPIT, NHPT, and BBT, abnormal behavior was defined if task performance was outside the 95^*th*^-percentile of a normative reference population. According to the ARAT, activity limitations were present if the score was below 55 [13]. All other conventional scales indicated the presence of impairments if the full score was not reached. Only participants with all conventional scales available were used. In total, 90% and 50% of all individuals showed impairment in at least one VPIT metric with the most affected and less affected side, respectively

**Fig. 2:**
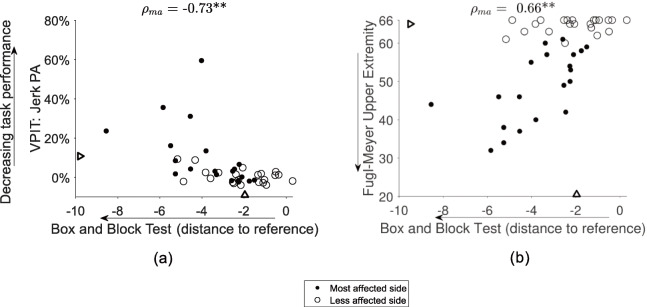
Example correlations between impairments (VPIT, Fugl-Meyer Upper Extremity) and activity limitations (Box and Block Test). The relationship of impairments and activity limitations was analyzed with Spearman correlations (*ρ*). Two pairs (**a**-**b**) were chosen for visualization purposes (all results in Table 2). Only data from the most affected side (*ρ*_*ma*_) and the first testing session was used for the correlation analysis. For both VPIT and conventional scales, triangles represent a cut-offs indicating the presence of sensorimotor impairments (VPIT, Fugl-Meyer Upper Extremity) and activity limitations (Box and Block Test). A slightly stronger relationship was observed between impairments and activity limitations for the VPIT metric than the Fugl-Meyer assessment. **indicates *p*-value below the Bonferonni corrected significance level. VPIT: Virtual Peg Insertion Test

Examples for the relationship between the VPIT metrics and conventional scales are visualized in Fig. 2 (all correlations in Table 2, confidence intervals in Table SM5). The following correlations were significant after Bonferroni correction: force rate SPARC transport with MOCA (*ρ*_*ma*_=-0.61**); jerk peg approach with BBT (*ρ*_*ma*_=-0.73**), ARAT (*ρ*_*ma*_=-0.65**), and NHPT (*ρ*_*ma*_=0.64**). Further, the correlations of the following conventional scales of impairments with the activity domain were significant after Bonferroni correction: FMA-UE with BBT (*ρ*_*ma*_=0.66**); MAS with BBT (*ρ*_*ma*_=-0.65**); FMA-UE with ARAT (*ρ*_*ma*_=0.82**); MAS with ARAT (*ρ*_*ma*_=-0.62**).

**Table 2:** Correlation between conventional scales and VPIT metrics for the most affected side

**Dependent variable**	**Spearman correlations** *ρ*_*ma*_ (*n* = 20)												
	**VPIT metrics**										**Conventional scales**		
	**Impairments in activity context**										**Impairments**		
	Log jerk TP	Log jerk RT	SPARC RT	Path length ratio TP	Path length ratio RT	Vel. max. RT	Jerk PA	GF num. peaks TP	GF rate SPARC TP	GF rate SPARC HA	FMA-UE	MAS	EmNSA
**Conventional scales**													
**Impairments**													
FMA-UE	-0.39	-0.40	-0.51*	-0.46*	-0.21	-0.14	-0.58*	0.37	0.16	-0.36			
MAS	0.51*	0.52*	0.59*	0.34	0.35	0.13	0.60*	-0.28	0.06	0.42			
MOCA	-0.11	0.12	0.08	-0.07	0.13	-0.40	-0.08	-0.17	-0.61**	-0.36			
EmNSA	-0.23	-0.28	-0.23	0.02	0.14	-0.13	-0.08	0.16	0.29	-0.04			
**Conventional scales**													
**Activities**													
BBT	-0.60*	-0.50*	-0.53*	-0.55*	-0.27	-0.18	-0.73**	0.20	-0.25	-0.58*	0.66**	-0.65**	0.17
ARAT	-0.27	-0.27	-0.43	-0.61*	-0.29	-0.07	-0.65**	0.30	0.05	-0.59*	0.82**	-0.62**	0.22
NHPT	0.37	0.44	0.39	0.49*	0.26	0.02	0.64**	-0.20	-0.11	0.40	-0.57*	0.60*	-0.44

MAS: Modified Ashworth Scale; MOCA: Montreal cognitive assessment; NHPT: Nine Hole Peg Test; EmNSA: Erasmus MC modifications to the Nottingham Sensory Assessment; BBT: Box and Block Test; ARAT: Action Research Arm Test; FMA-UE: Fugl-Meyer Assessment Upper Extremity; GF: grip force. SPARC: spectral arc length. num: number. vel: velocity. TP: transport. RT: return. PA: peg approach. HA: hole approach. The Bonferroni-corrected significance level was 0.05/13=0.0038 for the correlations with the BBT, ARAT, and NHPT, and 0.05/10=0.005 for all other conventional scales

Spearman correlation analysis was applied to analyze the relationship of conventional scales and VPIT metrics. Only data collected during the first testing session with the most affected body side was considered for this analysis. *indicates a *p*-value below 0.05 and **indicates a *p*-value below the Bonferroni-corrected significance level. Bonferonni correction was applied within each table row

### Test-retest reliability, measurement error, and learning effects of the VPIT metrics

**Fig. 3:**
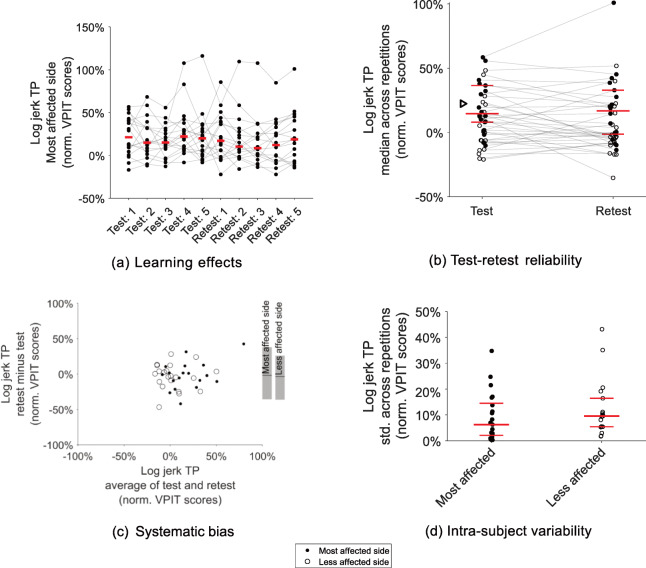
Clinimetric evaluation of the VPIT metrics: example log jerk transport. **a**) shows the behavior of all subjects across five repetitions of test and retest to visualize potential learning effects. **b**) informs on test-retest reliability by visualizing the median across those five repetitions for test and retest. The red line indicates the population median for the most affected side, the triangle corresponds to the 95^*th*^-percentile of the normative reference population, and shaded gray lines connect data from one subject. **c**) systematic bias was evaluated using a Bland-Altman plot (start and end of gray bars on the right indicate the 5^*th*^- and 95^*th*^-percentile). d) intra-subject variability was displayed through the standard deviation (std) within all ten repetitions of each subject. The example metric *log jerk transport* did not show strong learning effects, had high test-retest reliability, no systematic bias, and low intra-subject variability, therefore being defined as robust. TP: transport

Example visualization of the analyzed clinimetric properties can be found in Fig. 3 (all metrics in Figure SM4, SM5, and SM8). The test-retest reliability and measurement error of all metrics are summarized in Table 3. The metrics fullfilling all criteria for the quality of the clinimetric properties were the *log jerk transport* (ICC 0.89, SRD% 23.31, *η* -1.65), *log jerk return* (ICC 0.84, SRD% 28.56, *η* -4.85) and *force rate SPARC transport* (ICC 0.90, SRD% 20.49, *η* -5.02).

**Table 3:** Test-retest reliability: intra-class correlation (ICC) coefficients and smallest real differences (SRD)

Sensor-based metric	Test-retest reliability			
	Most affected side		Less affected side	
	*n* = 18		*n* = 21	
	ICC [CI]	SRD%	ICC [CI]	SRD%
Log jerk transport	**0.89** [0.83, 0.92]	**23.31**	**0.79** [0.69, 0.86]	30.79
Log jerk return	**0.84** [0.75, 0.89]	**28.55**	**0.89** [0.84, 0.93]	**25.31**
SPARC return	**0.81** [0.72, 0.88]	34.70	**0.87** [0.81, 0.91]	**27.91**
Path length ratio transport	0.58 [0.36, 0.72]	54.05	0.66 [0.50, 0.77]	52.38
Path length ratio return	0.49 [0.24, 0.66]	52.24	**0.84** [0.76, 0.89]	**29.09**
Velocity max return	**0.95** [0.92, 0.97]	**16.88**	**0.97** [0.93, 0.98]	**13.05**
Jerk peg approach	0.48 [0.22, 0.65]	94.55	**0.92** [0.88, 0.95]	**19.75**
Grip force rate num. peaks transport	**0.87** [0.80, 0.91]	**24.58**	**0.90** [0.84, 0.93]	**21.70**
Grip force rate SPARC transport	**0.90** [0.85, 0.94]	**20.49**	**0.89** [0.84, 0.93]	**21.43**
Grip force rate SPARC hole approach	**0.85** [0.72, 0.91]	34.20	**0.78** [0.67, 0.85]	41.39

The ICC (optimum at 1) describes the ability of a metric to discriminate between subjects across measurement sessions. The SRD% (optimum at 0%) describes a range of values for that the assessment cannot distinguish between measurement noise and an actual change in the underlying physiological construct. Bold ICC values represent acceptable test-retest reliability (i.e., above or equal 0.7). Bold SRD% indicate least strong measurement error (SRD% <30.3)

The metrics having insufficient (ICC <0.7) test-retest reliability were *path length ratio transport/return* and *jerk peg approach* for the most affected side and *path length ratio transport* for the less affected side. Systematic bias across test-retest session according to Bland-Altman plots was visible especially for *path length ratio transport/return* and *jerk peg approach*. The metrics *SPARC return*, *path length ratio transport/return*, *jerk peg approach*, and *grip force rate SPARC hole approach* for the most affected side as well as *log jerk transport*, *path length ratio transport*, and *grip force rate SPARC hole approach* for the less affected side did not pass the measurement error evaluation (SRD% >30.3).

On the most affected side, learning effects across test-retest were strong (*p*-value <0.05 and *η*>-6.35) for *path length ratio transport*, *velocity max. return*, *force rate num. peaks transport*, and *force rate SPARC hole approach* (Table SM6, Figure SM6). For the less affected side, learning effects were strong for *velocity max. return* and *force rate num. peaks transport* (Table SM6, Figure SM7).

## Discussion

The aim of this work was to evaluate the ability of the digital health metrics from a technology-aided assessment (VPIT) to characterize arm and hand sensorimotor impairments that are relevant for performing functional tasks, by evaluating their clinimetric properties in post-stroke individuals. The novelty of this work lies in the usage of a technology-aided assessment that has high clinical applicability and allows rapidly capturing movement and grip force patterns during a goal-directed, functionally relevant manipulation task requiring active lifting of the arm against gravity. Hence, we expected that the metrics provide a multi-dimensional, robust, and clinically applicable assessment of sensorimotor impairments in arm and hand with functional relevance. This hypothesis was evaluated in 30 chronic post-stroke subjects. Twenty-three of these, the ones with mild to moderate sensorimotor impairments and without strong cognitive deficits, were able to successfully complete the goal-directed manipulation task protocol with their most affected body side, thereby confirming previous reports about the feasibility of such tasks in individuals with mild to moderate neurological deficits [24, 32].

### Assessment of functionally relevant sensorimotor impairments with a technology-aided goal-directed manipulation task

The digital health metrics allowed identifying a high amount of individuals with impairments in the most affected (90%) and less affected (50%) side. This could only be achieved by considering multiple kinematic and kinetic metrics, thereby providing the envisioned multi-dimensional assessment of arm and hand sensorimotor deficits. Nevertheless, conventional assessments detected sensorimotor impairments in more post-stroke individuals (100% for most affected side with FMA-UE) than the digital health metrics, even though the latter have a more sensitive scale without ceiling effects. We argue that the reduced rate of detected impairments with the digital health metrics is caused by individuals compensating for certain impairments through the redundancy of the human motor apparatus [13, 41, 57]. These individuals can therefore still achieve normal performance during goal-directed tasks.

Moreover, the digital health metrics showed high significant correlations with the BBT and moderate significant correlations with the ARAT and NHPT. This suggests that the goal-directed manipulation task is able to describe sensorimotor impairments that are functionally relevant and especially related to the ability to repeatedly grasp and transport lightweight objects as well as dexterous finger manipulations. Indeed, it is intuitive that the goal-directed manipulation task is especially related to the BBT, given the similar movements that are required to complete the two tests. In addition, the correlations of the digital health metrics with the BBT and NHPT were slightly higher than the ones observed between conventional assessment of sensorimotor impairments (FMA-UE, MAS, EmNSA) and BBT and NHPT. We speculate that this slightly stronger relationship results from the digital health metrics being recorded during a functional task, whereas conventional assessments of impairments describe them in the absence of a functional context. For the ARAT, the correlations were considerably higher with the FMA-UE than with the digital health metrics. Compared to the technology-aided task, the FMA-UE and ARAT emphasize more on the ability to flex the shoulder, thereby explaining their strong relationship that has also been extensively reported in literature [11–13].

When relating these insights to the state of the art, it becomes obvious that only few technology-aided approaches quantify movements without arm weight support and also include object manipulations with the hand, which are especially important to linking impairments and activity limitations [24–27]. For example, Alt Murphy et al. showed a similar correlation, as reported herein, between movement smoothness and the ARAT for post-stroke subjects that performed a drinking task recorded with an optical motion capture system [24, 25]. Similarly, Johansson and Häger used an optical motion capture system for characterizing kinematics during a modified version of the NHPT and found high correlations between movement smoothness and the task completion time [27]. While these approaches are promising to relate sensorimotor impairments and activity limitations and further allow to study compensatory trunk movements, the solutions rely on a costly and time-consuming measurement setup with an optical motion capture system, thereby limiting their clinical applicability. Research towards more clinically applicable approaches has also been proposed, for example through the use of instrumented objects [28, 29] or the same robotic device as used by the VPIT [58, 59]. However, the former is limited in its ability to characterize movement patterns. In addition, the latter does not involve any precise object manipulations and relies on the regular handle of the robotic device that cannot record grip forces. Unsurprisingly, their reported correlations with the activity domain were considerably lower (multiple regression R ^2^ up to 13% for ARAT, which would correspond to a Pearson correlation of 0.36 for the univariate case) [58, 59]. Lastly, it is important to emphasize that approaches requiring arm-hand coordination and active lifting of the upper limb against gravity are especially tailored to individuals with mild to moderate neurological deficits, and diverging results can be observed in literature when considering subjects with more severe impairments [60–63]. This stems from such individuals typically having only a limited residual ability to use the hand, which makes the assessment of arm impairments sufficient to establish a link between impairments and activity limitations. Also, severely impaired individuals typically require arm weight support to perform goal-directed activities, thereby shadowing the influence of functionally relevant impairments such as weakness [19].

Hence, the proposed technology-aided assessment crystallizes as an interesting solution allowing a rapid (median 16.6 min with most affected side including instructions) and, relative to optical motion capture systems or exoskeletons, inexpensive (approx. 4000 USD hardware costs) assessment of sensorimotor impairments in arm and hand in individuals with mild to moderate disability. Moreover, the impairments detected with the technology-aided approach showed relevance for performing activities similar to the NHPT and BBT, which was enabled by the task involving precise manipulations, the absence of arm weight support, and the quantification of grip forces.

### Pathophysiological correlates of VPIT metrics and functional relevance of impairments

While conventional assessments (FMA-UE, MAS, EmNSA) capture sensorimotor impairments without functional context, it was still expected to observe moderate correlations between functionally relevant impairments and VPIT metrics. These correlated with the MAS and FMA-UE, which suggests that the metrics are sensitive to increased muscle tone and abnormal coupling of the shoulder, arm, and hand. While trends were visible for many metrics, the strongest ones were found for the metric *jerk peg approach*, which was also correlated most strongly to conventional scales of activity. This metric describes especially the precise coordination of movements and the release of grip forces that is required to insert a peg, which might be modulated by the integrity of the corticospinal tract [32, 64]. This idea is supported by the correlation with the FMA-UE and MAS, given that the abnormal coupling of joints is expected to be driven by corticospinal tract integrity, which can also contribute to increased muscle tone, depending on lesion location and severity [65–68]. However, these speculative statements require further validation, given that the correlations with the FMA-UE and MAS were not significant after Bonferroni correction, and that neurophysiological markers would be required for making strong conclusions. Also, a clear correlation of the FMA-UE with NHPT (not significant after Bonferroni), BBT, and ARAT was observed. This suggests the functional relevance of the ability to perform fractionated movements with single joints, as measured by the FMA-UE, that is expected to be modulated by corticospinal tract integrity. Alternatively, it might imply the co-occurrence of other functionally relevant impairments when the main neural transmission pathway is disrupted. Given that subjects often perform compensatory movements allowing to improve task performance in the presence of abnormal joint couplings [13, 41], we speculate that the latter option is not unlikely. In addition, we observed a reduced ability to perform goal-directed activities in individuals with increased muscle tone. These results are in line with literature, even though the clinical importance of spasticity post-stroke is subject to critical discussions [69, 70].

Somatosensory impairments, as assessed by the EmNSA, were not significantly correlated to any VPIT metrics and did not contribute to functional task performance in the conventional scales. Interestingly though, moderate correlations (significant before Bonferroni) were found for the *force rate SPARC hole approach* metric and the BBT and ARAT. Given that this metric characterizes grip force coordination and is expected to be influenced by sensory deficits [32], we speculate that these deficits might not have been captured by the clinical scale of sensory impairments that is well known to lack sensitivity [71].

The only VPIT metric being significantly correlated to the MOCA as a general descriptor of cognitive impairments was the force rate SPARC transport. This might result from a misunderstanding of the visual feedback provided by the task and the subsequent uncoordinated application of grip forces.

These results showing moderate correlations between conventional impairment scales and digital health metrics are in general in line with literature, even though the observed relationships are strongly context-dependent [17, 72–74].

### Clinimetric properties of the VPIT metrics

The clinimetric properties of the ten VPIT core metrics were previously positively evaluated in unaffected subjects [32]. Also, a first preliminary evaluation of the VPIT was done in post-stroke subjects [35]. However, this evaluation relied on a different measurement protocol and did not yet consider the recently introduced ten core metrics, which were selected by applying conservative and objective selection criteria [32]. Herein, we confirm the robustness of three VPIT core metrics, *log jerk transport* (ICC 0.89, SRD% 23.31, *η* -1.65), *log jerk return* (ICC 0.84, SRD% 28.56, *η* -4.85), and *force rate SPARC transport* (ICC 0.90, SRD% 20.49, *η* -5.02) in the most affected side of chronic post-stroke subjects. This implies that these metrics are highly reliable, have no strong measurement error, and are not showing strong learning effects. Based on these rather low measurement errors, the metrics are expected to be suitable for sensitively assessing sensorimotor impairments in a longitudinal manner [54, 55]. Given the previous validation, all ten metrics can still be used to detect the presence of sensorimotor impairments in cross-sectional studies [32]. Reasons why the metrics were more robust in neurologically intact than affected subjects might be the smaller sample size used for the analysis in this work as well as higher intra-subject variability in post-stroke subjects (Fig. 3 and SM8). This rather high variability might be because the VPIT allows heterogeneous task completion strategies and the haptic device being able to render only up to 3.3 N of haptic feedback, which can lead to an unstable haptic rendering of the virtual reality environment. Also, the variability might be influenced by a visuomotor transformation from the end-effector to the virtual reality environment that has to be learned throughout multiple repetitions of the task (Figure SM6), as also observed in other virtual reality-based assessments [75].

It is challenging to compare the clinimetric properties of the VPIT metrics to the ones extracted from other technology-aided assessments due to the context-dependence of metrics [17, 74]. Moreover, there is a lack of quality in the evaluation of technology-aided assessments and in-depth and thorough validation is only rarely implemented [17]. In the few cases where measurement error has been reported, its magnitude was again dependent on the assessment metric and platform, with overall mostly similar ranges (e.g., SRD of 13.2% to 95.0%) to the VPIT metrics [63, 76–80]. Compared to conventional assessments (e.g., FMA-UE measurement error of 7.9%; ARAT of 6.1%) [77, 81], the measurement errors of most technology-aided assessment metrics seem consistently elevated, even though comparisons are also challenged by the use of different SRD implementations. Nevertheless, we argue that this results from technology-aided assessments providing a multi-dimensional picture of the behavioral components underlying task performance, which makes them more susceptible to behavioral variability compared to the often ordinal outcome measures of conventional scales. Hence, we recommend researchers to thoroughly evaluate the clinimetric properties of technology-aided assessments and especially consider intra-subject variability as an important factor when designing assessment tasks. This is fundamental to fulfil the high expectations of the research community about technology-aided assessments providing more sensitive outcome measures than conventional scales.

### Limitations

The major limitation of this work is the limited amount of post-stroke participants included in the analysis, which reduces the generalizability of the results to other individuals that potentially show different impairment phenotypes. This also led to rather high confidence intervals (Table SM5) for the correlation analysis and emphasizes the need for further validation. Further, compensatory movements, for example by the trunk, were not captured by the end-effector based approach, but might be important to fully understand the relationship between impairments and activity limitations.

## Conclusions

This work provides evidence about the importance of technology-aided assessments that are considering precise goal-directed manipulations and grip forces without arm weight support, such as the VPIT. These approaches can enable a robust, sensitive, and objective way to assess arm and hand sensorimotor impairments that are functionally relevant in chronic post-stroke individuals with mild to moderate deficits. Further, the VPIT allowed implementing such an approach in a highly clinically applicable manner, by being rapidly applicable and, for a technology-aided assessment, inexpensive. This promises to better identify impairments with high functional relevance as therapy targets in clinical research and practice, which might ultimately contribute to optimizing the functional benefits of neurorehabilitation interventions.

In the future, it should be explored whether the assessment with the VPIT provides clinical benefits when used as a complementary source of information in clinical practice. Further, the presented results should be confirmed within large-scale trials, where structural neuroimaging markers together with clustering approaches should be used to fully unravel the pathophysiological correlates of digital health metrics.

## Supplementary information

**Additional file 1** Supplementary material.

## Abbreviations

ARAT: Action research arm test
BBT: Box and block test
Erasmus modified Nottingham sensory assessment; FMA-UE: Fugl-Meyer assessment upper extremity
GF: Grip force
HA: Hole approach
ICC: Intra-class correlation coefficient
MAS: Modified Ashworth scale
MOCA: Montreal cognitive assessment
Num: Number
PA: Peg approach
RT: Return
SRD: Smallest real difference
SPARC: Spectral arc length
TP: Transport
Vel: Velocity
VPIT: Virtual peg insertion test
