# Supplementary material for: Technology-aided assessment of functionally relevant sensorimotor impairments in arm and hand of post-stroke individuals
Source: J Neuroeng Rehabil. 2020 Sep 25;17:128. doi: 10.1186/s12984-020-00748-5 (PMC7517659; doi:10.1186/s12984-020-00748-5)
Supplement: Supplementary file 1 — Additional file 1 Supplementary material. [file 12984_2020_748_MOESM1_ESM.pdf]

Supplementary material for Kanzler et al., Technology-aided assessment of functionally relevant sensorimotor impairments in arm and hand of post-stroke individuals, 2020, JNER

Table SM1: **Demographic and clinical information for all post-stroke subjects.** FMA-UE: Fugl-Meyer Assessment Upper Extremity. ARAT: Action Research Arm Test. NHPT: Nine Hole Peg Test. BBT: Box and Block Test. MAS: Modified Ashworth Scale (sum across muscle groups). EmNSA: Erasmus modified Nottingham Sensory Assessment. MOCA: Montreal Cognitive Assessment.

ID	Age	Gender	Tested limb	Impaired limb	Dominant limb	Chronicity (weeks)	FMA-UE	ARAT	NHPT (s)	BBT (1/min)	MAS	EmNSA	MOCA	Has retest
1	67	Male	Right	Left	Right	112.98	66	57	23.25	43	0	40	25	1
2	55	Male	Left	Left	Right	91.25	54	56	33.25	50	0	33	27	1
2	55	Male	Right	Left	Right	91.25	66	57	21.85	69	0	40	27	1
3	55	Male	Left	Right	Right	108.63	65	57	22.82	50	0	40	25	1
3	55	Male	Right	Right	Right	108.63	49	55	29.28	45	3	39	25	1
4	52	Male	Left	Left	Right	147.74	55	52	35.36	40	2	40	21	1
4	52	Male	Right	Left	Right	147.74	65	57	20.99	59	0	40	21	1
5	73	Male	Left	Right	Right	48.14	62	-	-	-	0	38	27	0
6	69	Female	Right	Left	Right	46.29	61	57	20.32	40	0	39	22	1
7	67	Male	Left	Left	Right	130.43	50	-	-	-	2	39	14	0
7	67	Male	Right	Left	Right	130.43	66	-	-	-	0	40	14	0
8	40	Female	Left	Right	Right	41.71	56	45	-	-	0	39	27	0
8	40	Female	Right	Right	Right	41.71	49	49	-	-	1	38	27	0
9	71	Male	Left	Left	Left	242.71	40	35	196.69	27	7	31	28	1
9	71	Male	Right	Left	Left	242.71	65	57	15.03	54	1	40	28	1
10	59	Female	Left	Left	Right	235.14	50	47	17.7	56	1	40	28	1
10	59	Female	Right	Left	Right	235.14	66	57	12.57	65	0	40	28	1
11	88	Female	Left	Left	Right	89.14	37	39	42.17	30	3	37	26	0
11	88	Female	Right	Left	Right	89.14	63	-	14.33	56	0	39	26	0
12	69	Female	Left	Right	Right	31.57	63	57	19.81	38	0	40	23	1
12	69	Female	Right	Right	Right	31.57	44	39	49.16	19	2	40	23	1
13	59	Female	Left	Right	Right	104.86	66	57	21.5	55	0	39	28	0
13	59	Female	Right	Right	Right	104.86	57	56	21.63	56	1	40	28	0
14	50	Female	Right	Left	Right	260.71	64	-	-	-	0	40	29	0

Table SM1: **Continued.**

ID	Age	Gender	Tested limb	Impaired limb	Dominant limb	Chronicity (weeks)	FMA-UE	ARAT	NHPT	BBT	MAS	EmNSA	MOCA	Has retest
15	61	Male	Left	Right	Right	469.7	66	56	36.98	31	0	36	24	1
15	61	Male	Right	Right	Right	469.7	38	42	53.75	25	5	39	24	1
16	59	Male	Left	Left	Right	88.29	46	40	56.38	26	7	38	28	0
16	59	Male	Right	Left	Right	88.29	63	57	21.96	37	0	40	28	1
17	69	Male	Left	Left	Right	27.71	53	51	31.66	50	0	39	28	1
17	69	Male	Right	Left	Right	27.71	63	56	19.61	65	0	40	28	1
18	55	Male	Left	Left	Right	78.21	59	57	28.08	58	0	40	30	1
18	55	Male	Right	Left	Right	78.21	66	57	18.5	71	0	40	30	1
19	42	Male	Left	Left	Right	26.07	39	30	-	23	0	36	28	1
19	42	Male	Right	Left	Right	26.07	65	57	20.47	52	0	40	28	1
20	51	Female	Left	Right	Right	52.14	66	57	21.01	53	0	39	24	1
20	51	Female	Right	Right	Right	52.14	61	57	25.7	50	1	38	24	1
21	58	Male	Left	Right	Right	26.07	62	57	23.33	63	0	40	27	1
21	58	Male	Right	Right	Right	26.07	42	53	26	46	3	38	27	1
22	46	Male	Left	Left	Right	56.49	57	42	24.03	50	0	39	23	1
22	46	Male	Right	Left	Right	56.49	66	57	23.09	66	0	40	23	1
23	76	Male	Left	Right	Right	147.74	66	55	39.73	40	0	40	21	1
23	76	Male	Right	Right	Right	147.74	60	54	29.19	39	1	40	21	1
24	53	Female	Left	Right	Right	160.77	66	57	22.99	65	0	40	26	1
24	53	Female	Right	Right	Right	160.77	58	55	20.67	59	0	38	26	1
25	62	Male	Left	Right	Right	790.83	66	57	19.58	73	0	40	27	1
25	62	Male	Right	Right	Right	790.83	34	33	154	25	3	38	27	1
26	62	Male	Left	Right	Right	56.49	64	57	24.6	60	1	40	29	1
26	62	Male	Right	Right	Right	56.49	46	43	86	23	2	35	29	1
27	69	Male	Left	Right	Right	52.14	60	54	22.69	48	0	40	26	1
27	69	Male	Right	Right	Right	52.14	32	34	59.63	27	3	39	26	1

Table SM2: **Confidence intervals for correlation between conventional scales and VPIT metrics for the most affected side.** Spearman correlation analysis was applied to analyze the relationship of conventional scales and VPIT metrics. Ninety-five percent confidence intervals were constructed via Fisher's z-transform and reported as 'lower bound, upper bound'. Only data collected during the first testing session with the most affected body side was considered for this analysis.MAS: Modified Ashworth Scale; MOCA: Montreal cognitive assessment; NHPT: Nine Hole Peg Test; EmNSA: Erasmus MC modifications to the Nottingham Sensory Assessment; BBT: Box and Block Test; ARAT: Action Research Arm Test; FMA-UE: Fugl-Meyer Assessment Upper Extremity; GF: grip force. SPARC: spectral arc length. num: number. vel: velocity. TP: transport. RT: return. PA: peg approach. HA: hole approach.

**Dependent variable**	**Spearman correlations ρ_{ma}** n = 20												
	**VPIT metrics** **Impairments in activity context**										**Conventional scales** **Impairments**		
	Log jerk TP	Log jerk RT	SPARC RT	Path length ratio TP	Path length ratio RT	Vel. max. RT	Jerk PA	GF num. peaks TP	GF rate SPARC TP	GF rate SPARC HA	FMA-UE	MAS	EmNSA
**Conventional scales**													
**Impairments**													
FMA-UE	-0.71, 0.06	-0.72, 0.05	-0.78, -0.09	-0.75, -0.02	-0.6, 0.25	-0.55, 0.32	-0.81, -0.19	-0.08, 0.7	-0.3, 0.57	-0.69, 0.1			
MAS	0.08, 0.78	0.1, 0.78	0.19, 0.82	-0.12, 0.68	-0.11, 0.69	-0.33, 0.54	0.22, 0.82	-0.64, 0.19	-0.4, 0.49	-0.03, 0.73			
MOCA	-0.53, 0.35	-0.34, 0.53	-0.38, 0.5	-0.5, 0.38	-0.33, 0.54	-0.71, 0.05	-0.5, 0.38	-0.57, 0.3	-0.83, -0.24	-0.69, 0.09			
EmNSA	-0.61, 0.24	-0.64, 0.18	-0.61, 0.23	-0.43, 0.46	-0.32, 0.55	-0.54, 0.34	-0.5, 0.38	-0.31, 0.56	-0.18, 0.65	-0.48, 0.41			
**Conventional scales**													
**Activities**													
BBT	-0.82, -0.21	-0.77, -0.08	-0.79, -0.11	-0.8, -0.14	-0.63, 0.2	-0.58, 0.29	-0.89, -0.43	-0.27, 0.59	-0.62, 0.22	-0.82, -0.19	0.3, 0.85	-0.85, -0.3	-0.3, 0.57
ARAT	-0.64, 0.19	-0.63, 0.2	-0.73, 0.01	-0.83, -0.22	-0.65, 0.17	-0.5, 0.38	-0.85, -0.3	-0.16, 0.66	-0.4, 0.48	-0.82, -0.2	0.6, 0.93	-0.83, -0.25	-0.25, 0.6
NHPT	-0.09, 0.7	0, 0.74	-0.07, 0.71	0.06, 0.77	-0.21, 0.63	-0.43, 0.46	0.27, 0.84	-0.59, 0.27	-0.53, 0.35	-0.05, 0.72	-0.81, -0.17	0.22, 0.83	-0.74, 0.01

Table SM3: **Quantification of learning effects.** A linear regression model was used to test, per metric, whether a systematic improvement, indicative of learning effects, between test and retest was present. The slope η was normalized relative to the range of observed values. Bold entries indicate metrics without strong learning effects (effect non-significant or η >-6.35).

Sensor-based metric	Learning effects (η)	
	Most affected side n = 18	Less affected side n = 21
Log jerk transport	**-1.65**	**-4.00**
Log jerk return	**-4.85**	**-6.67**
SPARC return	**-8.10**	**-6.67**
Path length ratio transport	**-7.68**	**0.80**
Path length ratio return	-13.28	**-4.23**
Velocity max. return	-8.86	-8.90
Jerk peg approach	**-9.92**	**3.21**
Grip force rate num. peaks transport	-10.16	-7.16
Grip force rate SPARC transport	**-5.02**	**-4.64**
Grip force rate SPARC hole approach	-18.90	**-5.46**

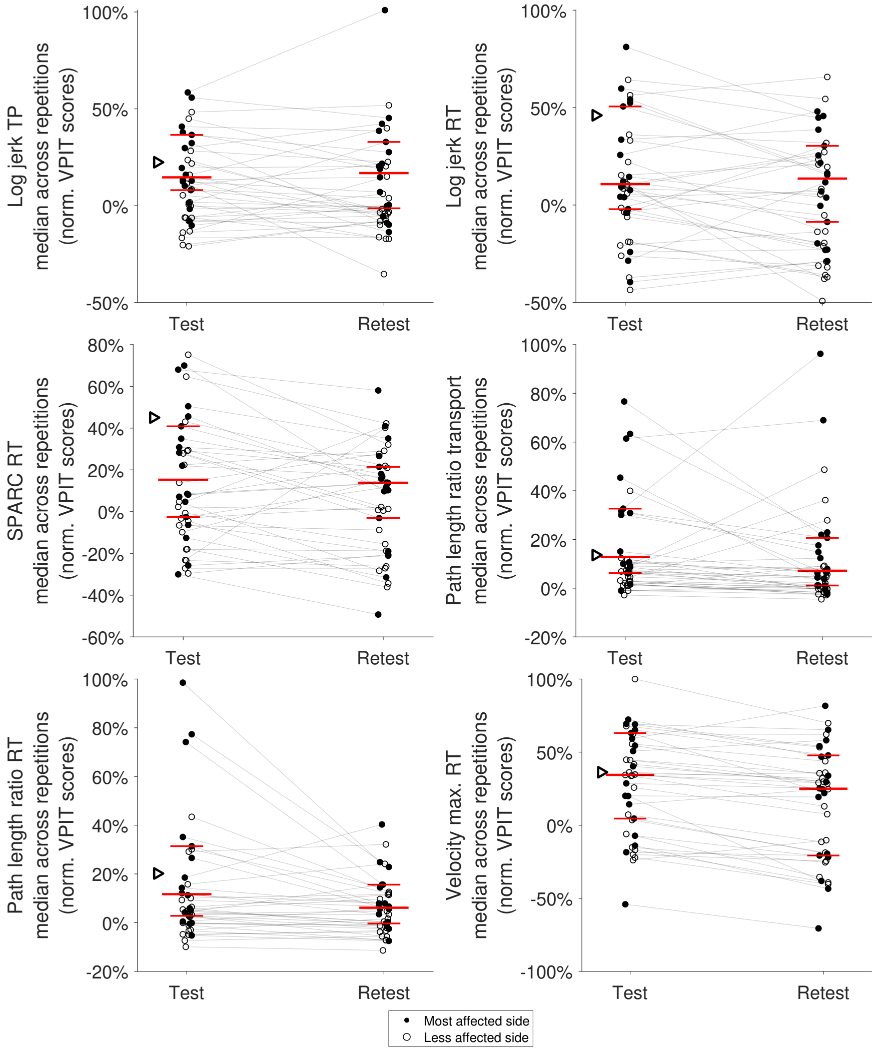

Figure SM1: **Test and retest scores for all VPIT metrics.** The task performance level decreases with increasing VPIT scores. Additionally, 0% represents the median of an unimpaired reference population and 100% the task performance of the worst neurological subject in the VPIT database. The long red horizontal bar indicates the population median for the most affected side. The shorter red horizontal bars represent the 25th- and 75th-percentile. The black triangle represents the 95th-percentile of the unimpaired reference population. Pre- and post-measurements of a single subject and body side are connected with a gray line.

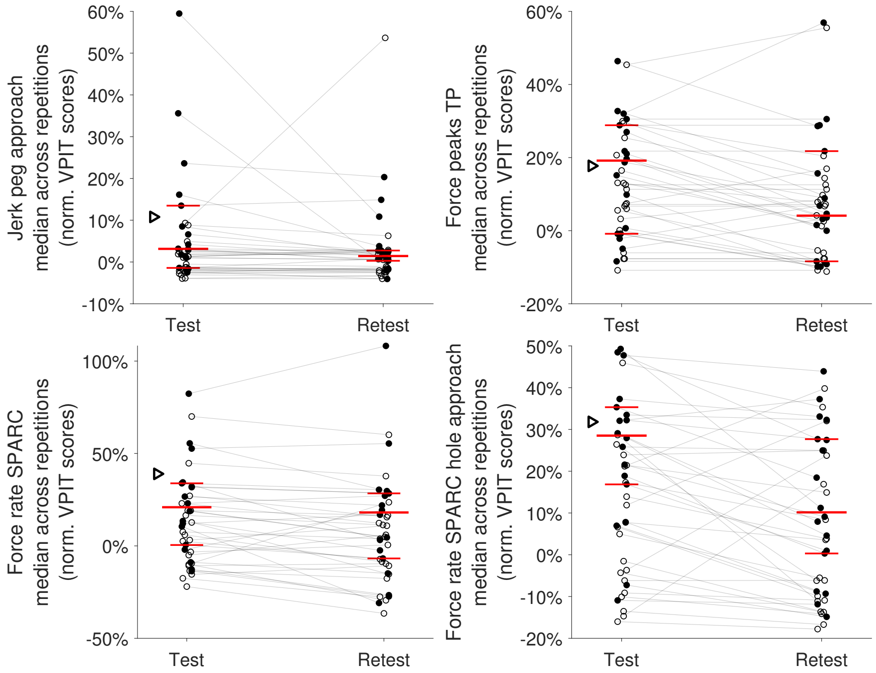

Figure SM1: **Continued.**

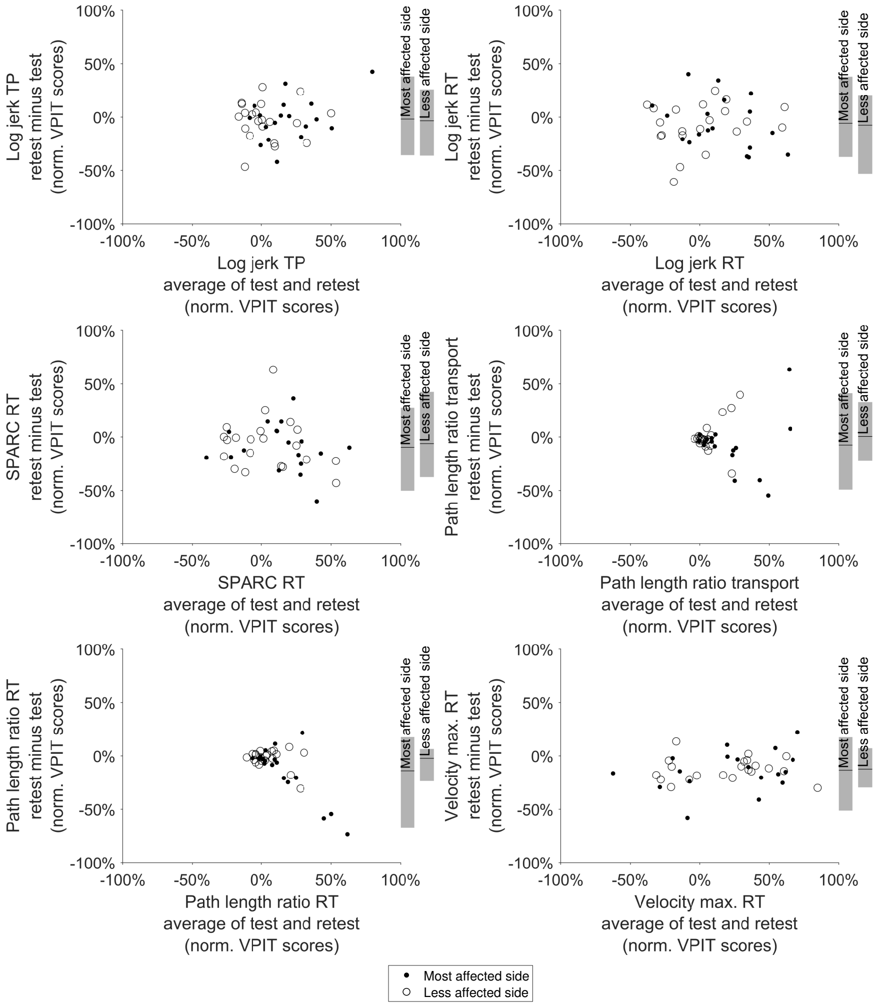

Figure SM2: **Bland-Altman plots for the sensor-based metrics of the VPIT.** The vertical axis represents the difference between the test and retest measurement, whereas the horizontal axis represents their average. The dashed horizontal line represents ideal behavior (i.e., zero difference between measurements). Further, the solid black horizontal bars and the shaded gray areas represent the median, 5th and 95th-percentile, respectively, for subjects tested on the most and less affected side. TP: transport. RT: return.

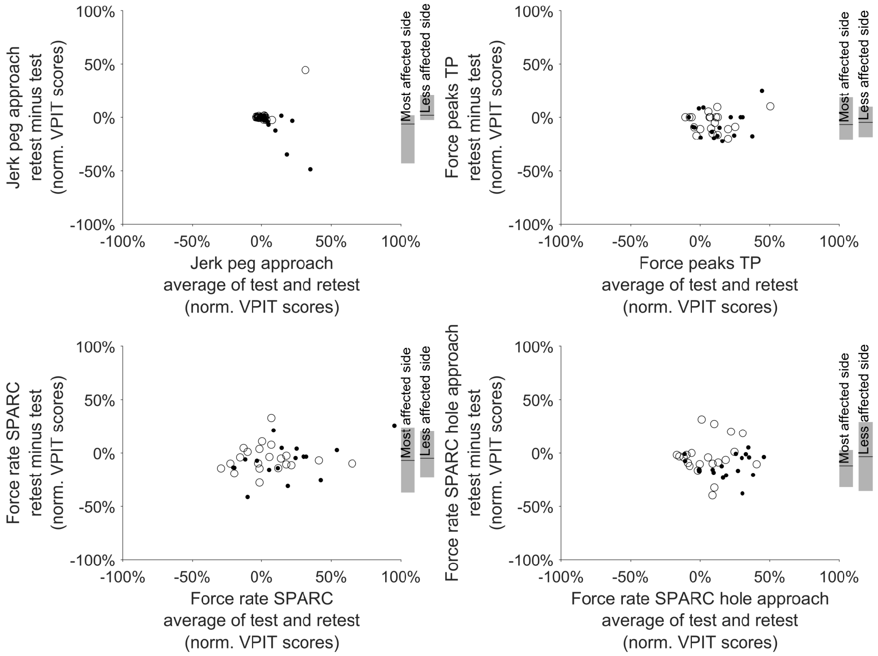

Figure SM2: **Continued.**

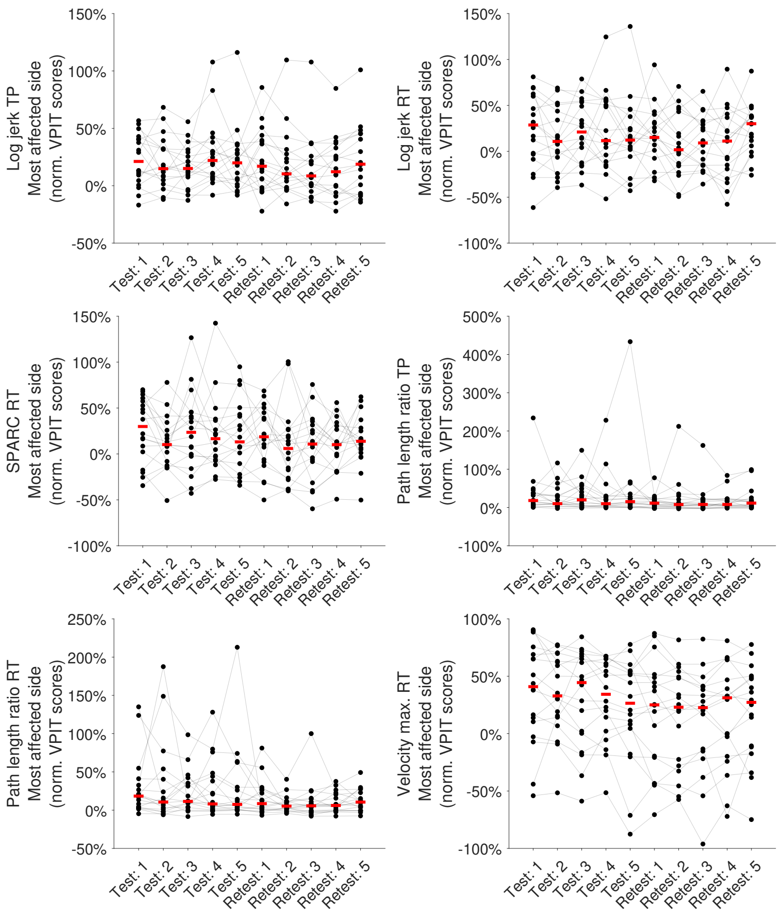

Figure SM3: **Learning effects in the VPIT metrics for the most affected side.** The behavior of all subjects across five repetitions of test and retest is visualized to identify potential learning effects. Gray horizontal lines connect the data of one individual. The red line indicates the median across subjects. TP: transport. RT: return. SPARC: spectral arc length.

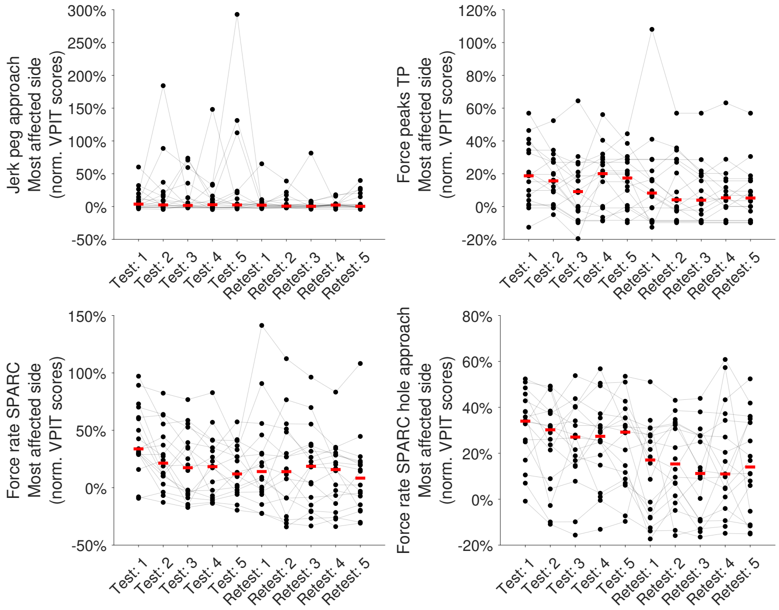

Figure SM3: **Continued.**

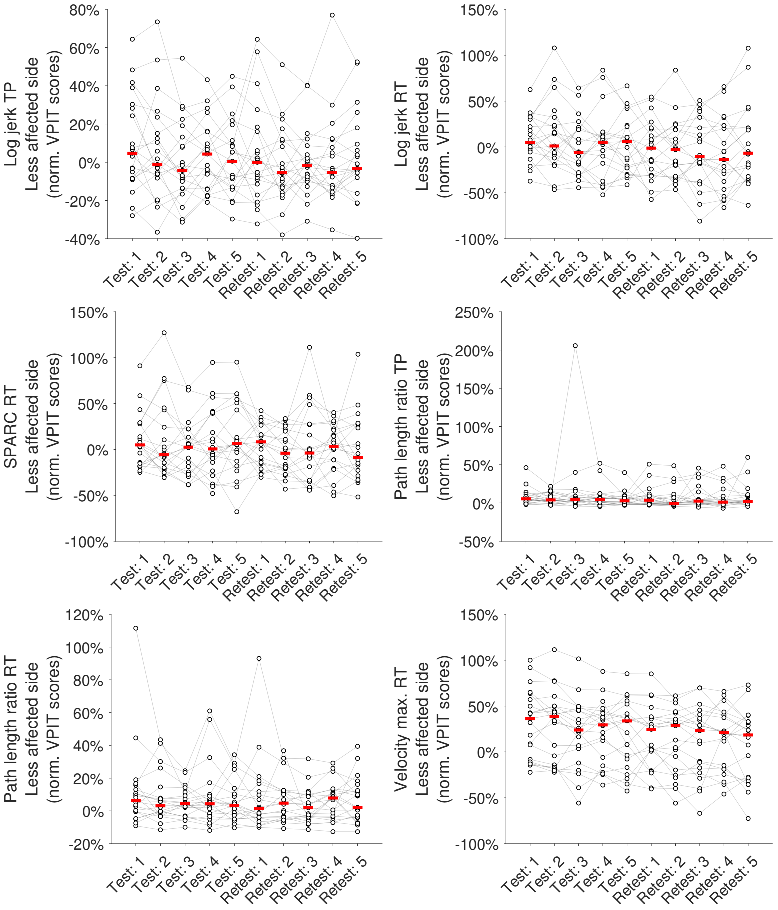

Figure SM4: **Learning effects in the VPIT metrics for the less affected side.** The behavior of all subjects across five repetitions of test and retest is visualized to identify potential learning effects. Gray horizontal lines connect the data of one individual. The red line indicates the median across subjects. TP: transport. RT: return. SPARC: spectral arc length.

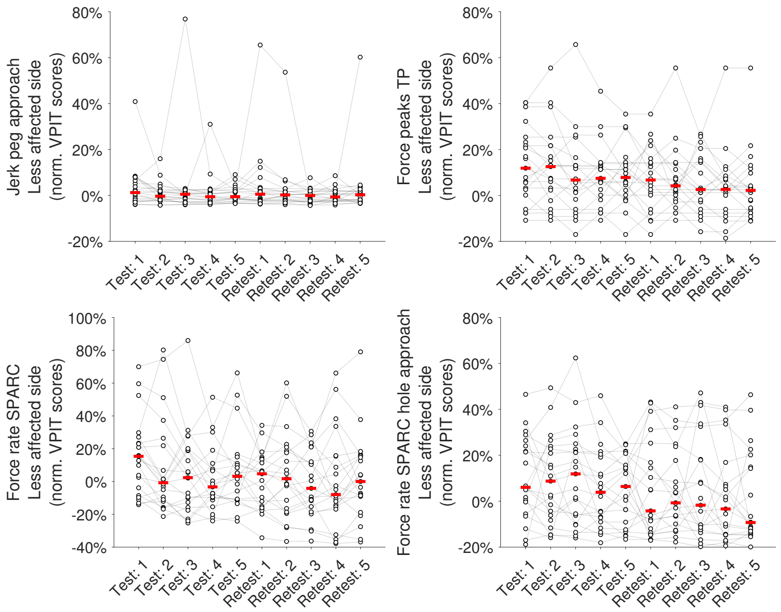

Figure SM4: **Continued.**

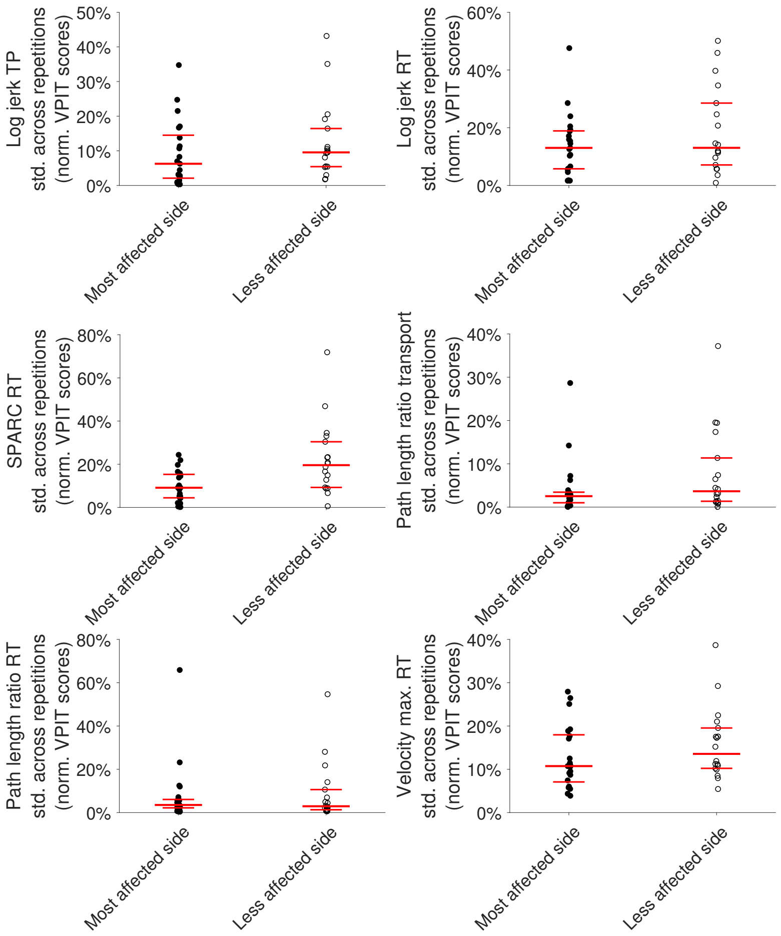

Figure SM5: **Intra-subject variability of the VPIT metrics.** The standard deviation within the ten repetitions of the VPIT of each subjects was visualized. The longest red line indicates the population median, whereas the shorter red lines indicate the 25th and 75th-percentiles. TP: transport. RT: return. SPARC: spectral arc length.

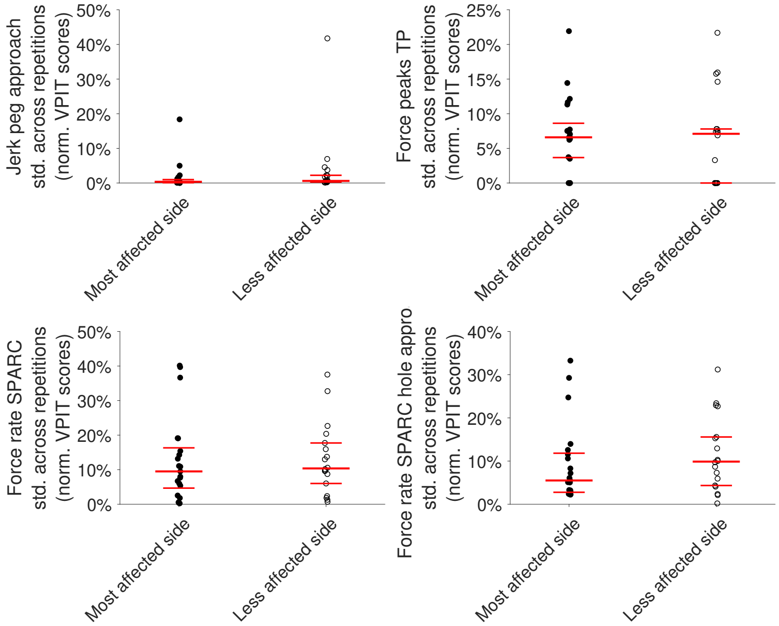

Figure SM5: **Continued.**
